# Risk factors of in-hospital death in patients with acute ST elevation myocardial infarction

**DOI:** 10.1007/s11739-020-02338-8

**Published:** 2020-04-28

**Authors:** Yong Li

**Affiliations:** Emergency and Critical Care Center, Beijing Anzhen Hospital, Capital Medical University, Beijing, 100029 China

**Keywords:** Coronary disease, ST elevation myocardial infarction, In-hospital mortality, Killip classification

## Background

Coronary heart disease remains the leading cause of mortality [1]. Prevention of in-hospital death is a crucial step in improving prognosis of patients with ST elevation myocardial infarction (STEMI). We want to investigate the risk factors of in-hospital death.

## Methods

### Source of data

Totally 9668 patients with acute STEMI in Beijing Anzhen Hospital, Capital Medical University from January 2002 to August 2019.

Inclusion criteria: (1) patient hospitalized with STEMI; (2) age of more than 18 years.

We established the diagnosis of acute myocardial infarction (AMI) and STEMI base on fourth universal definition of myocardial infarction [2].

Exclusion criteria: none.

### Evaluation and diagnosis of in-hospital death

All causes for in-hospital death is defined as cardiac or non-cardiac death during hospitalization.

### Predictors

We selected 11 predictor variables for inclusion in our prediction rule. They were shown in Table 1. PCI = percutaneous coronary intervention, CABG = coronary artery bypass grafting. Atrial fibrillation is defined as all type of atrial fibrillation during hospitalization. Atrioventricular block is defined as all type of atrioventricular block during hospitalization.

**Table 1:** Clinical characteristics of patients with in-hospital death and in-hospital survivors

Characteristic [lower limit, upper limit]	In-hospital deaths (*n* = 188)	In-hospital survivors (*n* = 9480)	Odds Ratio	*P* >|*Z*|	95% CI
Age (year, *x* ± *s*) [21, 91]	71 ± 12	59 ± 12	1.1	< 0.001	1.084–1.116
Man *n* (%) 0 = no, 1 = yes	119 (63.3)	7602 (80.2)	0.426	0.001	0.315–0.576
History of hypertension *n* (%) 0 = no, 1 = yes	122 (64.9)	5352 (56.5)	1.426	0.021	1.054–1.929
History of diabetes *n* (%) 0 = no, 1 = yes	64 (34)	2864 (30.2)	1.192	0.258	0.879–1.617
History of myocardial infarction *n* (%) 0 = no, 1 = yes	29 (15.4)	763 (8)	2.084	< 0.001	1.393–3.117
History of PCI *n* (%) 0 = no, 1 = yes	15 (8)	771 (8.1)	0.979	0.939	0.575–1.668
History of CABG *n* (%) 0 = no, 1 = yes	3 (1.6)	53 (0.6)	2.884	0.077	0.893–9.314
Killip classification *n* (%) 0 = no, 1 = yes					
Killip I	8 (4.3)	4936 (52.1)	0.041	< 0.001	0.02–0.083
Killip II	25 (13.3)	3429 (36.2)	0.271	< 0.001	0.178–0.413
Killip III	31 (16.5)	628 (6.6)	2.783	< 0.001	1.878–4.126
Killip IV	124 (66)	490 (5.2)	35.548	< 0.001	25.94–48.712
Atrial fibrillation *n* (%) 0 = no, 1 = yes	35 (18.6)	449 (4.7)	4.601	< 0.001	3.149–6.723
Atrioventricular block *n* (%) 0 = no, 1 = yes	18 (9.6)	249 (2.6)	3.925	< 0.001	2.376–6.484
Underwent PCI during hospitalization *n* (%) 0 = no, 1 = yes	51 (27.1)	7328 (77.3)	0.109	< 0.001	0.079–0.151

### Statistical analysis

We followed the methods of Li et al. 2019 [3].

## Results

### Participants and predictors of in-hospital death

Totally 188 patients had in-hospital death (in-hospital death group) and 9480 patients had no in-hospital death (control group). The results are shown in Table 1.

### Predictors of in-hospital death

Eight variables (age, gender, history of myocardial infarction, history of hypertension, Killip classification, atrial fibrillation, atrioventricular block, and underwent PCI during hospitalization) were significant differences in the two groups of patients (*p *< 0.05). After application of backward variable selection method, three variables (underwent PCI, age, and Killip classification) remained as significant independent predictors of in-hospital death. Results are shown in Tables 2 and 3.

**Table 2:** Predictor of in-hospital death obtained from multivariable logistic regression models (odds ratio)

In-hospital death	Odds Ratio	Std. Err	*Z*	*P* >|*Z*|	95% CI
Age	1.05	0.008	5.99	< 0.001	1.033–1.066
Underwent PCI during hospitalization	0.343	0.065	− 5.67	< 0.001	0.237–0.497
Killip II	3.079	1.164	2.97	0.003	1.467–6.461
Killip III	10.61	3.992	6.28	< 0.001	5.076–22.181
Killip IV	64.715	21.981	12.28	< 0.001	33.257–125.929
_Cons	0.0002	0.0001	− 13.20	< 0.001	0.00006–0.0008

**Table 3:** Predictor of in-hospital death obtained from multivariable logistic regression models (Coef)

In-hospital death	Coef	Std. Err	*Z*	*P* >|*Z*|	95% CI
Age	0.048	0.008	5.99	< 0.001	0.033–0.064
Underwent PCI during hospitalization	− 1.069	0.188	− 5.67	< 0.001	− 1.438– − 0.699
Killip II	1.125	0.378	2.97	0.003	0.384–1.866
Killip III	2.362	0.376	6.28	< 0.001	1.625–3.099
Killip IV	4.17	0.34	12.28	< 0.001	3.504–4.836
_Cons	− 8.426	0.639	− 13.20	< 0.001	− 9.677– − 7.174

We drew the receiver operating characteristic curve. The area under the receiver operating characteristic curve was 0.94 ± 0.007, 95% CI = 0.926–0.954.

### Study limitations

This is a single-center experience. Some patients were enrolled > 10 years ago, thus their treatment may not conform to current standards and techniques.

## Discussion

We investigated the predisposing factors of in-hospital death. A frequency of in-hospital death was 1.9% (188/9668). Killip classification is an independent risk factor of in-hospital death. In our study, patients with Killip class IV were at 64.7 higher risk of in-hospital death than patients with Killip class I–III. Not underwent PCI is an independent risk factor of in-hospital death. Patients who do not get successful reperfusion are at higher risk of early complications and death [4]. Age is an independent risk factor of in-hospital death. Older patients have more comorbidities and are less likely to receive reperfusion therapy [5, 6]. Elderly patients are also at particular risk of bleeding [4].

## Conclusions

Age, not underwent PCI during hospitalization, and Killip classification are independent risk factors for predicting in-hospital death in patients with acute STEMI.

## Electronic supplementary material

Below is the link to the electronic supplementary material.

Supplementary file1 (CSV 321 kb)
